# Potential Mechanisms of the Sparing of Atopic Dermatitis in the Diaper Region: A Scoping Review

**DOI:** 10.1177/12034754221088533

**Published:** 2022-03-22

**Authors:** Aria Jazdarehee, Jason Lee, Richard Lewis, Ilya Mukovozov

**Affiliations:** 1 8166 Department of Medicine, University of British Columbia, Vancouver, BC, Canada; 2 Department of Dermatology and Skin Science, University of British Columbia, Vancouver, BC, Canada; 3 Kamloops Dermatology, Kamloops, BC, Canada

**Keywords:** Eczema, atopic dermatitis, childhood, infants, diaper, nappy area

## Abstract

Atopic dermatitis (AD) is a chronic, inflammatory skin condition commonly affecting infants with notable sparing of the diaper region. Though sources anecdotally attribute this sparing to the physical barrier formed by the diaper and the subsequent retention of moisture, urine, sweat and feces, no studies have formally investigated the factors contributing to this sparing phenomenon. We performed a scoping literature review to investigate the factors involved in sparing of AD in the diaper region, namely humidity, scratching, urine, sweat, feces, and microbiome composition. A total of 130 papers met the inclusion criteria, and extracted data were analyzed in an iterative manner. Increased local humidity facilitates protective changes at the cellular level and offsets transepidermal water loss. Exposure to urea from both sweat and urine may contribute to improved moisturization of the skin through its natural humectant properties and ability to modulate gene expression. Introduction of flora in feces contributes to the generation of protective immune responses and outcompetes growth of pathogens such as *Staphylococcus aureus*. Finally, diapers physically prevent scratching, which directly interrupts the itch-scratch cycle classically implicated in AD. Our study reviews factors that may contribute to the sparing of AD in the diaper region in infants. A limitation to our findings is that the studies reviewed here explore the impacts of these factors on AD broadly, and not explicitly in the diaper region. Additional studies investigating this may further our understanding of AD pathogenesis and contribute to the development of effective therapeutics.

## Introduction

Atopic dermatitis (AD) is a chronic inflammatory dermatosis characterized by skin barrier dysfunction resulting in pruritic, eczematous lesions.^1^ The disease impacts all ages and ethnicities, and has the highest burden among skin diseases worldwide.^1,2^ The pathophysiology of AD is complex and involves an interplay of genetic, immune, and environmental factors which collectively result in skin barrier dysfunction.^3^ The exact mechanisms of AD remain under investigation and there remain no curative treatments of the disease.^1^ The presentation of AD is variable and depends on many factors, including age.^4^ Among infants, AD commonly presents with red, scaly lesions on the face, neck, and extensor surfaces.^5^ Despite the variation in clinical presentation, AD consistently spares the diaper region in infants. Given the pronounced sparing of AD within the diaper region, investigation of the mechanisms that underlie this sparing may elucidate therapeutic targets for the larger population affected by AD.

The sparing of AD within the diaper region has been noted in the literature,^1-4,6-9^ but studies have not explored the factors contributing to this phenomenon. Rather, studies have anecdotally suggested reasons for why this notable sparing occurs. Theories include protection due to the trapped moisture within the occlusive diaper region^7,8,10^ and prevention of scratching due to the overlying diaper.^8,10^

With this in mind, we conducted a scoping literature review to explore factors which may be implicated with AD’s sparing of the diaper region. This review evaluates the evidence supporting the existing theories of sparing, and explores other factors not previously considered. By elucidating the underlying mechanisms, we aim to provide direction for future therapeutic development.

## Methods

Given the lack of formal studies investigating AD’s sparing of the diaper region specifically, a scoping literature review was selected for broad assessment of evidence relevant to this topic. Unlike systematic reviews, which critically appraise individual studies, a scoping review allows for systematic mapping of research done in a broad context.^11^ Like systematic reviews, scoping reviews require structured, comprehensive searches to produce reproducible results.^11^ The protocol for this scoping review was drafted in accordance with the Preferred Reporting Items for Systematic Reviews and Meta-analysis Extension for Scoping Reviews (PRISMA-ScR).^12^

### Search Strategy, Study Eligibility Criteria, and Study Selection

Prior to the formal literature review, we conducted preliminary informal searches to determine potential areas of interest that were relevant to the sparing of AD within the diaper region. Based on these initial findings, we selected four factors to explore further: (i) moisture and humidity, (ii) urine and sweat, (iii) feces and microbiota, and (iv) prevention of scratching. After identifying these factors, we carried out four independent literature searches to further assess the relevance of these factors in the context of AD. Because studies have not been done exploring the effects of these factors within the diaper region specifically, we explored the effects of these factors on AD broadly.

The four independent searches were done using the MEDLINE database through the OVID interface. Search strategies were developed in collaboration with a medical librarian.

Studies were deemed eligible for inclusion if they were published in peer-reviewed journals, written in English, and published between 1980 and 2020. Both human trials and animal studies were considered. Opinion articles and commentaries were excluded to minimize bias.

Final search results were transferred onto COVIDENCE software (www.covidence.org) for title, abstract, and full-text screening.^13^ Two reviewers (AJ, JL) independently evaluated titles, abstracts, and then full-texts to identify relevant studies. Disagreements were resolved by consensus and through discussion with the senior reviewer (IM).

### Data Extraction and Synthesis

Two reviewers (AJ, JL) independently extracted data from eligible studies using a standardized extraction form that included title, authors, year of publication, study population, and key findings. Disagreements were resolved by consensus and through discussion with the senior reviewer (IM). All reviewers evaluated the extracted data and worked together to synthesize the available evidence in an iterative manner. Findings were synthesized into the four factors outlined below.

## Results

### Moisture and Humidity

Diapers are occlusive in nature and trap moisture effectively. This has been suggested to contribute to sparing of AD within the diaper.^14^ We searched for the effects of moisture and humidity on AD and yielded 737 results, of which 45 were deemed relevant for review. Our results suggest that the trapped moisture within the diaper region may improve skin barrier function, thereby reducing water loss and preventing entry of irritants, which may protect against the development of AD.

It is well-established that moisturizers are beneficial for the dry, damaged skin that is characteristic of AD. As such, moisturizers are commonly used in the management of AD to improve skin barrier function.^15^ Mechanistically, the stratum corneum (SC) is responsible for providing the protective barrier of the skin, which both retains moisture and protects skin from invading pathogens and irritants.^16^ At low environmental humidity, the SC is fragile and breakable due to rigidity of the keratin filaments that fill the corneocytes which make up the SC.^17^ With increased relative humidity, the glycine and serine amino acids within the keratin filaments undergo conformational change, resulting in improved molecular mobility.^18^ Accordingly, increased humidity improves skin barrier function, thereby decreasing transepidermal water loss (TEWL) and penetration of allergens and pathogens which may exacerbate AD.^19,20^

While high humidity is known to benefit skin barrier function and improve AD symptoms, low humidity is known to aggravate AD symptoms.^19^ Reports of AD exacerbations are often noted during the winter months of lower environmental humidity.^21^ Low environmental humidity is associated with epidermal hyperplasia, mast cell degranulation, and histological evidence of inflammation implicated with AD.^22^ Conversely, increased environmental humidity has been shown to reverse inflammation and epidermal abnormalities.^19^

The conditions within the diaper are characterized by increased moisture and humidity, due to the occlusive nature of the garment that prevents moisture from escaping.^23^ Accordingly, we suggest that this moist, humid environment may facilitate the changes to the stratum corneum that improves skin barrier function and decreased pathogen entry, through the mechanisms described above.

### Urine and Sweat

Given the sequestering of urine and sweat within the diaper, we hypothesize that these fluids may contribute to the sparing of AD. We searched for the effects of urine and sweat on AD and yielded 360 results, of which 35 were deemed relevant for review. We found that urine and sweat in the diaper may play an active role in maintenance of skin barrier function and protection against pathogens, thus protecting the local skin from AD.

Both urine and sweat contain urea, which is a humectant known to have beneficial effects on skin hydration. Though an irritant at supraphysiological doses, at physiological doses, urea improves skin hydration and water retention, which maintains a fluid SC and reduces TEWL.^18,24^ Urea has been shown to independently provide the same benefits on SC fluidity as increased humidity alone.^18^ Urea-based creams are based on these advantages and have been widely used since the 1940s.^25^

Emerging evidence suggests that urea also plays an active role in maintaining epidermal structure and function. Physiological doses of urea upregulate expression of genes involved in keratinocyte differentiation, lipid synthesis, and production of antimicrobial peptides (AMPs).^26^ These include genes which encode for transglutaminase 1, involucrin, filaggrin, loricin, as well as two AMPs, human β-defensin 2 and LL-37, which are both expressed in lower levels among those with acute and chronic AD.^26,27^

Focusing specifically on sweat, studies suggest that sweat may play a protective role in AD. Though initially thought to be an exacerbator of AD, disturbance of normal sweating function may be a driver of allergic skin inflammation.^28^ Those with chronic AD produce sweat droplets which are smaller in size and fewer in number compared to normal controls.^29^ Sweat contains many moisturizers including lactate, sodium, potassium, as well as urea, whose beneficial impacts on skin barrier function are described above.^30^ Sweat also carries fluid from the dermis to the SC, and it has been proposed that the moisture content contained within sweat could exceed TEWL due to the skin barrier defects associated with AD.^28^ Sweat may also provide an additional defense from pathogens, as dermcidin is an AMP produced exclusively by sweat glands.^31^ These additional defenses may protect against colonization of pathogens implicated in AD pathogenesis, such as *Staphylococcus aureus*, which is further described in the next section.

### Feces and Microbiota

Given the recent focus on the impacts of the microbiota on disease processes, we hypothesized that exposure to fecal flora may have a protective role against AD within the diaper region. We searched for the effects of feces and microbiota on AD and yielded 262 results, of which 41 were deemed relevant for review. We found that exposure of feces and associated commensals within the diaper region may play a role in promoting tolerant immune responses and outcompeting pathogens associated with the development of AD.

It is well established that a heavy, diverse microbiota beneficially directs the body’s immune responses, particularly towards tolerant regulatory *T* cell responses and away from inflammatory Th2 responses.^32^ Th2 responses are implicated with perturbed skin barrier function through downregulation of antimicrobial peptides, increased expression of serine proteases, and downregulation of keratinocyte differentiation.^33-35^ With relation to AD, those affected by the disease have less microbial diversity than healthy controls,^36^ and differences in microbiota early in life are predictive of later AD development.^35^

In addition to promoting protective immune responses, a diverse microbiota may also serve to discourage growth of pathogens known to exacerbate AD. One such pathogen whose effects on AD pathogenesis have been well-researched is *S. aureus*, which has been implicated with skin barrier disruption, immune system dysfunction, and alteration of the microbiome in AD patients.^37^ This is achieved, in part, through the bacterium’s ability to produce superantigens, which can directly bind major histocompatibility complex (MHC) II and generate polyclonal *T* cell responses with heavy cytokine release, thereby aggravating dermatitis.^37^ The presence of diverse commensals is, in turn, associated with production of AMPs and short-chain fatty acids which discourage pathogen growth.^38,39^ Accordingly, individuals with AD have less microbial diversity with enhanced *S. aureus* abundance.^36^ However, reintroduction of commensals to those with AD has been shown to decrease pathogen abundance and improve disease symptoms.^40^

It is this finding of disease regression with restored microbiota that underlies the rationale for topical probiotic use in AD management. Of particular note is a 2018 study which demonstrated that topical application of *Roseomonas mucosa* resulted in decreased disease severity while also decreasing steroid requirements and *S. aureus* burden.^41^ Other studies have demonstrated that a simple exposure to bacterial lysates also decreases disease severity and improves skin barrier function.^42^

These findings need to be considered within the specific environment of the diaper. Though urine and feces may independently protect against AD, it has been suggested that the alkaline urine may activate fecal lipases, ureases, and proteases, which may irritate the skin and predispose to diaper dermatitis.^43^

### Prevention of Scratching

It has been suggested that AD’s sparing of the diaper region is facilitated by prevention of skin scratching due to the overlying diaper.^44^ We searched for the effects of scratching on AD and yielded 643 results, of which 15 were deemed relevant for review. We found that the diaper may effectively interrupt the itch-scratch cycle and prevent further skin damage and inflammation that results from scratching.

Itch is a major symptom of AD that also contributes to skin damage and disease progression through a relentless cycle termed the itch-scratch cycle.^45^ Damaged skin activates specific sensory nerve terminals in the skin, which transmits the signal to the central nervous system, initiating a scratch response.^45,46^ Scratching further damages skin and results in inflammatory responses, further aggravating dermatitis.^45^ Resulting inflammatory responses further perpetuate this cycle through production of histamine and interleukin-31, which are key inducers of itch that act on independent neural pathways.^46,47^

Consistently, mouse models have demonstrated that clipping of hind toenails not only improve AD disease severity but also inhibit development of dermatitis.^48^ Mice that were prevented from scratching demonstrated improved skin barrier function as measured by TEWL and decreased serum immunoglobulin E levels, as well as decreased evidence of skin inflammation on histological analysis.^48^

## Discussion

Our study identified four factors that may be involved in the sparing of AD within the diaper region: (i) moisture and humidity, (ii) urine and sweat, (iii) feces and exposure to microbiota, and (iv) prevention of scratching.

AD is a disease characterized by skin barrier dysfunction whose causes are multifactorial and include *FLG* mutations, physical damage from scratching, and microbial dysbiosis.^1^ Disruptions in skin barrier function result in increased permeability and TEWL, reduced water composition, and altered lipid composition.^1,49-51^ Our results suggest that the factors within the diaper region may both protect against development of skin barrier dysfunction and also ameliorate the consequences of skin barrier dysfunction.

Specifically, the diaper prevents scratching and exposure to microbial commensals protects against microbial dysbiosis by outcompeting pathogens, both of which protect against the development of skin barrier dysfunction. Increased humidity, urine, and sweat actively influence skin barrier structure and function to strengthen the skin barrier, thereby reducing TEWL and preventing entry of irritants which may exacerbate AD.

Our study gives attention to AD’s notable sparing of the diaper region and explores the role of various factors that may be involved. A limitation of our study is that the factors discussed are explored broadly and not within the context of the diaper region, given that studies focusing on the diaper region do not exist. Consequently, the results of this scoping review are extrapolated and theoretical. Given the complexity of AD pathophysiology and unique environmental conditions of the diaper, studies should be done to understand how these factors influence AD development within the diaper region. Further understanding of the mechanisms behind the sparing of AD may provide direction for future therapeutic development and ultimately lower the global burden of this disease.

## Supplemental Material

Figure S1 - Supplemental material for Potential Mechanisms of the Sparing of Atopic Dermatitis in the Diaper Region: A Scoping Review Click here for additional data file. Supplemental material, Figure S1, for Potential Mechanisms of the Sparing of Atopic Dermatitis in the Diaper Region: A Scoping Review by Aria Jazdarehee, Jason Lee, Richard Lewis and Ilya Mukovozov in Journal of Cutaneous Medicine and Surgery
