# Supplementary material for: Potential Mechanisms of the Sparing of Atopic Dermatitis in the Diaper Region: A Scoping Review
Source: J Cutan Med Surg. 2022 Mar 22;26(4):398–403. doi: 10.1177/12034754221088533 (PMC9361425; doi:10.1177/12034754221088533)
Supplement: Figure S1 - Supplemental material for Potential Mechanisms of the Sparing of Atopic Dermatitis in the Diaper Region: A Scoping Review [file sj-docx-1-cms-10.1177_12034754221088533.docx]

**Supplementary Tables and Figures**

**Identification of studies via databases and registers**

Records removed *before screening*:

Duplicate records removed (n = 0)

Records marked as ineligible by automation tools (n = 0)

Records removed for other reasons (n = 0)

Records identified from MEDLINE:

Databases (n = 737)

Registers (n = 0)

**Identification**

Records screened

(n = 737)

Records excluded

(n = 692)

Reports sought for retrieval

(n = 45)

Reports not retrieved

(n = 0)

**Screening**

Reports assessed for eligibility

(n = 45)

Reports excluded:

Irrelevant outcomes (n = 33)

Foreign language (n = 3)

Full text not available (n = 0)

Studies included in review

(n = 9)

Reports of included studies

(n = 9)

**Included**

**Supplemental Figure 1:** PRISMA diagram corresponding to moisture and humidity literature search.

**Identification of studies via databases and registers**

Records removed *before screening*:

Duplicate records removed (n = 0)

Records marked as ineligible by automation tools (n = 0)

Records removed for other reasons (n = 0)

Records identified from MEDLINE:

Databases (n = 360)

Registers (n = 0)

**Identification**

Records screened

(n = 360)

Records excluded

(n = 325)

Reports sought for retrieval

(n = 35)

Reports not retrieved

(n = 0)

**Screening**

Reports assessed for eligibility

(n = 35)

Reports excluded:

Irrelevant outcomes (n = 26)

Foreign language (n = 0)

Full text not available (n = 0)

Studies included in review

(n = 9)

Reports of included studies

(n = 9)

**Included**

**Supplemental Figure 2:** PRISMA diagram corresponding to urine and sweat literature search**.**

**Identification of studies via databases and registers**

Records removed *before screening*:

Duplicate records removed (n = 0)

Records marked as ineligible by automation tools (n = 0)

Records removed for other reasons (n = 0)

Records identified from MEDLINE:

Databases (n = 262)

Registers (n = 0)

**Identification**

Records screened

(n = 262)

Records excluded

(n = 221)

Reports sought for retrieval

(n = 41)

Reports not retrieved

(n = 0)

**Screening**

Reports assessed for eligibility

(n = 41)

Reports excluded:

Irrelevant outcomes (n = 28)

Foreign language (n = 0)

Full text not available (n = 1)

Studies included in review

(n = 12)

Reports of included studies

(n = 12)

**Included**

**Supplemental Figure 3:** PRISMA diagram corresponding to feces and microbiota literature search**.**

**Identification of studies via databases and registers**

Records removed *before screening*:

Duplicate records removed (n = 1)

Records marked as ineligible by automation tools (n = 0)

Records removed for other reasons (n = 0)

Records identified from MEDLINE:

Databases (n = 643)

Registers (n = 0)

**Identification**

Records screened

(n = 642)

Records excluded

(n = 627)

Reports sought for retrieval

(n = 15)

Reports not retrieved

(n = 0)

**Screening**

Reports assessed for eligibility

(n = 15)

Reports excluded:

Irrelevant outcomes (n = 9)

Foreign language (n = 0)

Full text not available (n = 1)

Studies included in review

(n = 5)

Reports of included studies

(n = 5)

**Included**

**Supplemental Figure 4:** PRISMA diagram corresponding to scratching literature search.
